# Microbead Encapsulation Strategy for Efficient Production of Extracellular Vesicles Derived From Human Mesenchymal Stem Cells

**DOI:** 10.1002/jev2.70053

**Published:** 2025-04-16

**Authors:** Jiayi Tan, Yunxia Hu, Lijuan Zheng, Zheng Zheng, Mali Fu, Haiying Peng, Shaohua Ma

**Affiliations:** ^1^ Tsinghua Shenzhen International Graduate School (SIGS) Tsinghua University Shenzhen China; ^2^ Institute of Biopharmaceutical and Health Engineering (iBHE), Tsinghua Shenzhen International Graduate School (SIGS) Tsinghua University Shenzhen China; ^3^ Key Lab of Industrial Biocatalysis Ministry of Education Shenzhen China; ^4^ Key Lab of Active Proteins and Peptides Green Biomanufacturing of Guangdong Higher Education Institutes Tsinghua Shenzhen International Graduate School Shenzhen China; ^5^ Shenzhen Maternity and Child Healthcare Hospital Shenzhen China; ^6^ General Hospital of the Southern Theater Command of the Chinese People's Liberation Army Guangzhou China

**Keywords:** 3D microbeads, extracellular vesicles, large‐scale production, mesenchymal stem cell, microfluidic printing

## Abstract

Human mesenchymal stem cell‐derived extracellular vesicles (hMSC‐EVs) have shown great potential in tissue repair and regeneration. However, their scalable production and functional quality are still limited by current expansion technologies. In this study, we propose a production technology for hMSC‐EVs based on three‐dimensional (3D) microbead culture, which enhances the secretory behaviour of hMSC. Fixed number of MSCs were encapsulated in Matrigel at appropriate densities and printed into 3D microbeads by the custom automated microfluidic bead‐jet printing technique. Compared with 2D culture group, EVs derived from 3D hMSC microbead had smaller size and increased yield by 20‐fold, and the actin depolymerisation of the cell may be an important mechanism for enhancing EV secretion. Further analysis confirmed that the EVs derived from 3D hMSC microbead exhibited enhanced angiogenic and proliferative capabilities, which promoted the viability and tube‐forming capacity of human umbilical vein endothelial cells (HUVEC). In conclusion, this automated microfluidic microbead encapsulation technology increased the yield and therapeutic effect of hMSC‐EVs and provides a platform for scalable EV production of regenerative therapies.

## 1 Introduction

Mesenchymal stem cells (MSCs), as a type of pluripotent stem cells which possess strong proliferative, immunomodulation and tissue repair abilities, are emerging as a promising and safe therapeutic cell therapy for disease treatment (Pittenger et al. 2019). The available evidence indicates that the therapeutic effects of MSCs are primarily attributable to their paracrine effects (Fontaine et al. 2016). Extracellular vesicle (EV) is a type of nanoscale membrane vesicles actively secreted by living cells, which carry biomolecules such as proteins, RNA and DNA from the parent cell and play a crucial role in intercellular communication (Giebel et al. 2017; Vizoso et al. 2017). Given that human MSC‐derived EVs (hMSC‐EVs) retain the functions of the original cell, including pro‐angiogenesis, anti‐inflammation and immunomodulation, and that they have low immunogenicity and minimal risk of tumour formation, the direct use of hMSC‐EVs has been proposed as a new cell‐free therapy (Williams et al. 2023; Bunpetch et al. 2017; Park et al. 2019).

However, the scalable production and functional quality of hMSC‐EVs remain limited by current expansion technologies (Debbi et al. 2022; Lui and Leung 2022). Most commonly, hMSC‐EVs are produced through 2D cell culture (culture dish or T‐flask) combined with ultracentrifugation purification (Miceli et al. 2019; Kim et al. 2021). Although 2D cell culture is relatively easy to operate, the surface area of the culture plane is limited and requires frequent passaging. Thus, this strategy is labour‐intensive and time‐consuming. To address these limitations, 3D cell culture technology has been explored, providing cells with growth conditions closer to their natural physiological state and enhanced cell‐cell communication, thus better‐maintaining cell morphology, function and secretory behaviour compared with traditional 2D cell culture (Follin et al. 2016; Kouroupis and Correa 2021).

Recently, researchers have developed several 3D culture methods to increase EV yields (Rafiq et al. 2016; Yuan et al. 2022; Raghav et al. 2022; Kurogi et al. 2021). However, there are some shortcomings in existing 3D culture methods. For example, bioreactors require complex equipment and operating conditions, and cells may suffer from an insufficient supply of nutrients and oxygen during high‐density culture, affecting cell growth and EV secretion. In addition, the material selection and structural design of 3D‐printed bioscaffolds also have an impact on EV yield and function. Differences in the biocompatibility and mechanical properties of different materials may lead to an unstable cell growth environment, which in turn affects EV secretion and function. Therefore, the development of more efficient and scalable 3D culture technologies is important to achieve the large‐scale production of hMSC‐EVs.

This study aimed to develop a technique for the production of hMSC‐EVs based on three‐dimensional microbead cultures in order to improve the yield and functional quality of EVs. Based on our group's previous work, a microfluidic‐based bead‐jet printing system has been designed, which is capable of automatically using Matrigel to print uniform hMSC microbeads (Jiang et al. 2020; Xu et al. 2021). Within this platform, hMSCs of suitable density are encapsulated, and we speculate that the microbead niche will provide an appropriate 3D environment for hMSCs to maintain both growth and secretory behaviours. The potential impact of 3D cellular architecture on hMSC‐EVs was comprehensively investigated, including cytoskeleton organisation, EV production quantification, EV functional tests and miRNA profiles analysis, which gave insights into how 3D microbead culture affects the secretion, content and functions of hMSC‐EVs. Overall, this 3D microbeads encapsulation technology increased the yield and therapeutic effect of hMSC‐EVs and provides a platform for scalable EV production of regenerative therapies.

## 2 Result

### 2.1 Formation of hMSC Microbeads

**FIGURE 1:**
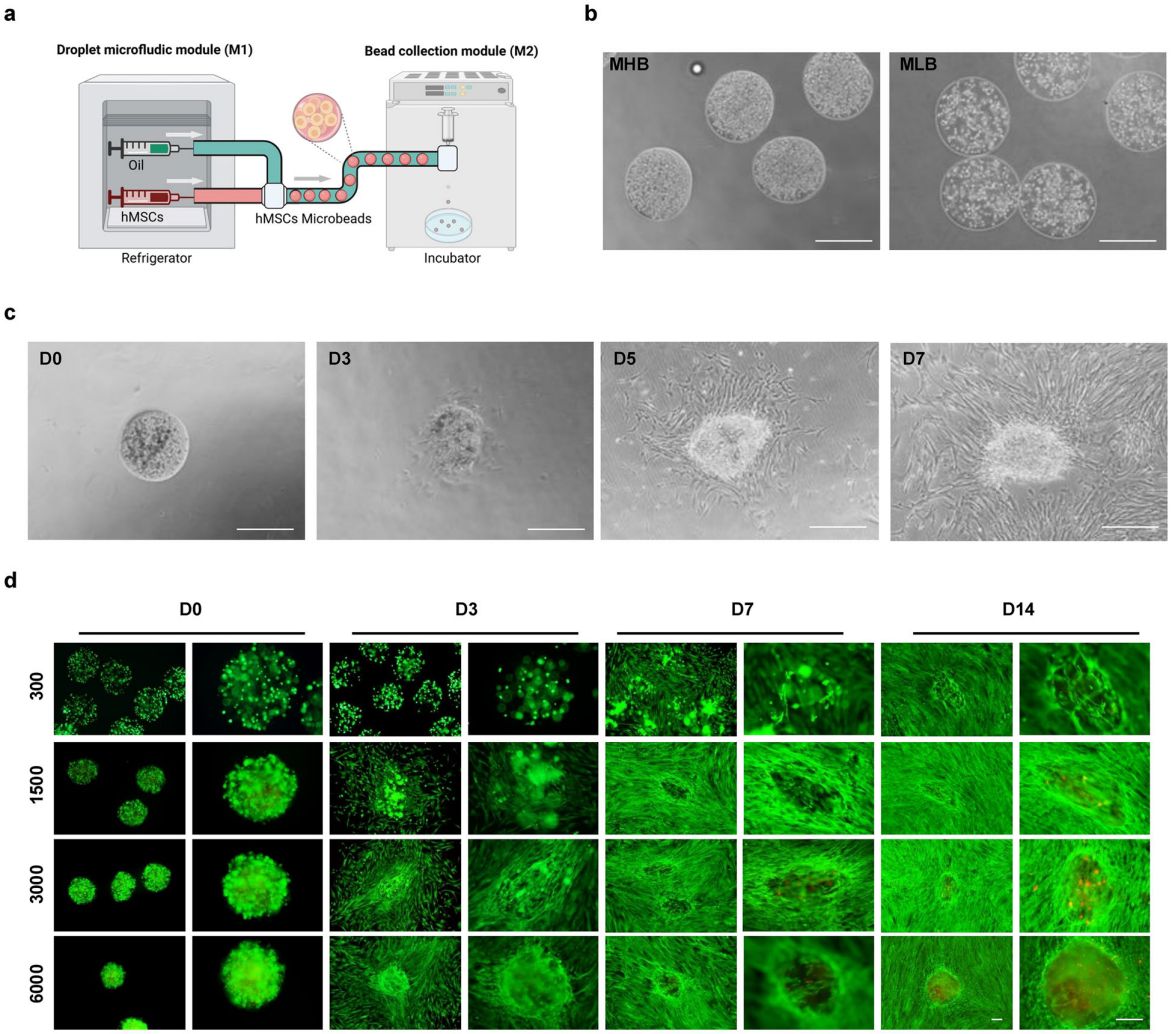
Characterisation and comparison of hMSCs derived from microbeads and 2D groups. (a) Schematic illustration of hMSCs microbead fabrication using microfluidic‐based bead‐jet printing system. (b) Bright‐field images of hMSC microbeads with high (1500 cells/bead) and low (300 cells/bead) cell density printed with microfluidic system at D0. Scale bar, 500 µm. (c) Microscopic images of high‐density hMSCs in Matrigel microbeads at D0, D3, D5 and D7. Scale bar, 500 µm. (d) AM/PI staining images of hMSCs microbeads from MLB and MHB groups at D0, D3 and D7 and D14. Scale bar, 200 µm.

Continuous and scalable formation of size‐controlled hMSC‐microbeads was accomplished using a microfluidic‐derived bead‐jet printing system (Figure 1a) designed from our previous work (Jiang et al. 2020; Xu et al. 2021; Cao et al. 2022). Specific microbead printing and production details can be found in the METHOD section.

Although the hMSC from the 2D culture/control group shows the morphology of fibroblast‐like cells and spindle‐shaped appearance. hMSCs culturing in Matrigel microbeads proliferated internally to form compact aggregates after being printed (Figure 1b). During Days 1–3, hMSC microbeads will gradually attach to the culture plate. From Day 3, these cells gradually grow out of the microbead scaffolds by modifying bead morphology and degrading the scaffolds. Later, hMSCs grew with adherence to achieve 70%–80% confluence (Figure 1c).

To test hMSC behaviour under different conditions, cell growth was tracked using live/dead staining by encapsulating 300, 1500, 3000 and 6000 cells per microbead (Figure 1d). The high cell density group exhibited a significantly higher proliferation rate compared with low cell density groups. However, 3000 and 6000 cells per microbead densities group showed more dead cells in the centre of hMSC‐microbeads on Day 14 (Figure 1d).

### 2.2 Comparison of EVs Derived From Microbeads and 2D Groups

**FIGURE 2:**
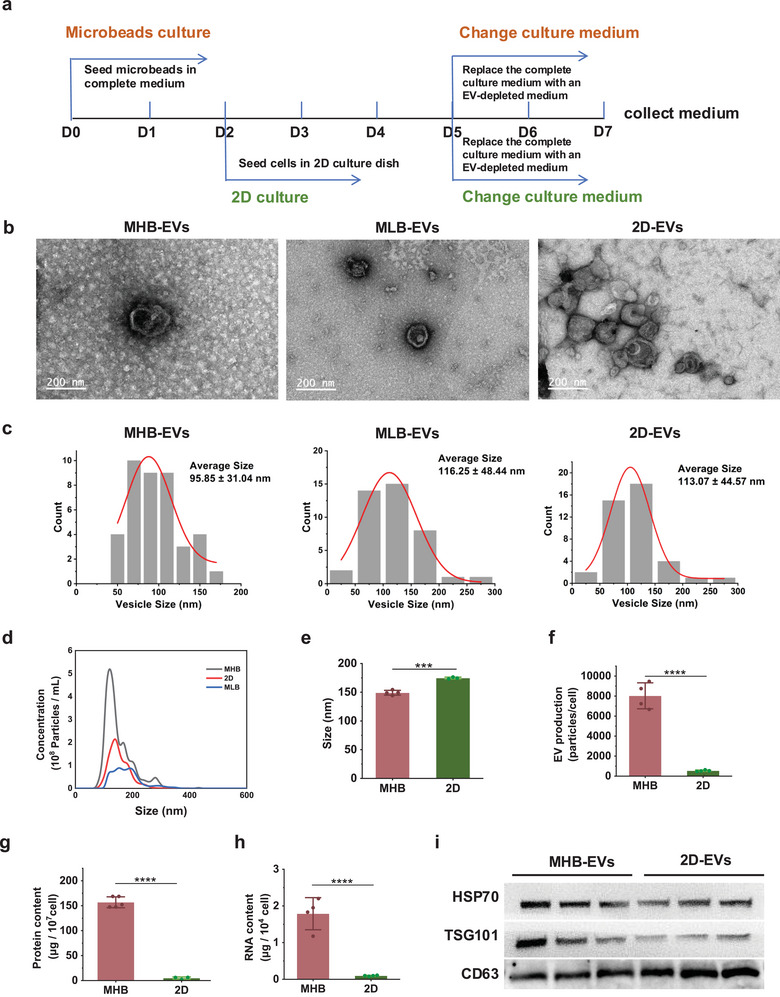
Characterisation and comparison of hMSC‐EVs derived from microbeads and 2D groups. (a) The experimental workflow of EV collection and isolation. (b) Representative TEM image of hMSCEVs. Scale bar, 200 nm. (c) Size distribution and concentration of hMSC‐EVs measured by NTA. (d) Comparison of size distribution and concentration results measured by NTA of hMSC‐EVs. (e and f) Average diameter and normalised particle concentration of hMSC‐EVs based on NTA results. (g and h) Protein and RNA content in hMSC‐EVs. (i) Western blot analysis of marker proteins (Hsp70, TSG101 and CD63) in hMSC‐EVs. *p < 0.05; **p < 0.01; ***p < 0.001; ****p < 0.0001.

To investigate the impact of 3D microbead cellular architecture on the production and functions of hMSC‐EVs, hMSCs were cultured in Matrigel‐low density‐bead (MLB, 300 cells/bead), Matrigel‐high density‐bead (MHB, 1500 cells/bead) and 2D conditions. The experimental flow of EV collection and isolation was built (Figure 2a). hMSCs in the microbead group were initially cultured in a complete culture medium for 5 days. In 2D groups, hMSCs were seeded on Day 2 and cultured in complete condition medium until Day 5. Next, the complete condition medium in all groups was replaced with EV‐depleted condition medium and culture for another 2 days. Then, EVs were collected from the medium of three groups by ultracentrifugation.

Transmission electron microscopy (TEM) was performed for EV morphology. TEM images showed round, lipid bi‐layered vesicular structures of EVs (Figure 2b). Nanoparticle tracking analysis (NTA) was performed to explore the particle size distribution (Figure 2c–e). More than 90% of the collected EVs were distributed between 50 and 250 nm. According to the NTA, EVs derived from the MHB group were smaller than 2D group, which is probably due to the 3D culture conditions affecting the cell morphology, cell‐cell interactions and biosynthesis and secretion processes of EVs (Mo et al. 2018). NTA analysis also showed that MHB group produced 14.96 times more EVs than 2D group (Figure 2f). The EV production efficiency was validated by normalised EV‐RNA and EV‐protein quantification (Figure 2g,h). Similarly, compared to 2D group, RNA quantification showed an 18.49‐fold increase, whilst protein quantification indicated a 30‐fold boost in EV production in MHB group. The differences in RNA and protein content between the two groups may be due to variations in EV cargo, which suggest a substantial improvement (approximately 15–20 fold) in EV production. At last, three typical EV markers (Hsp70, TSG101 and CD63) in the 2D‐EVs and MHB‐EVs groups were also validated by western blot (Figure 2i).

### 2.3 Microbead Culture Increases EV Production by Decreasing Actin Polymerisation of hMSCs

**FIGURE 3:**
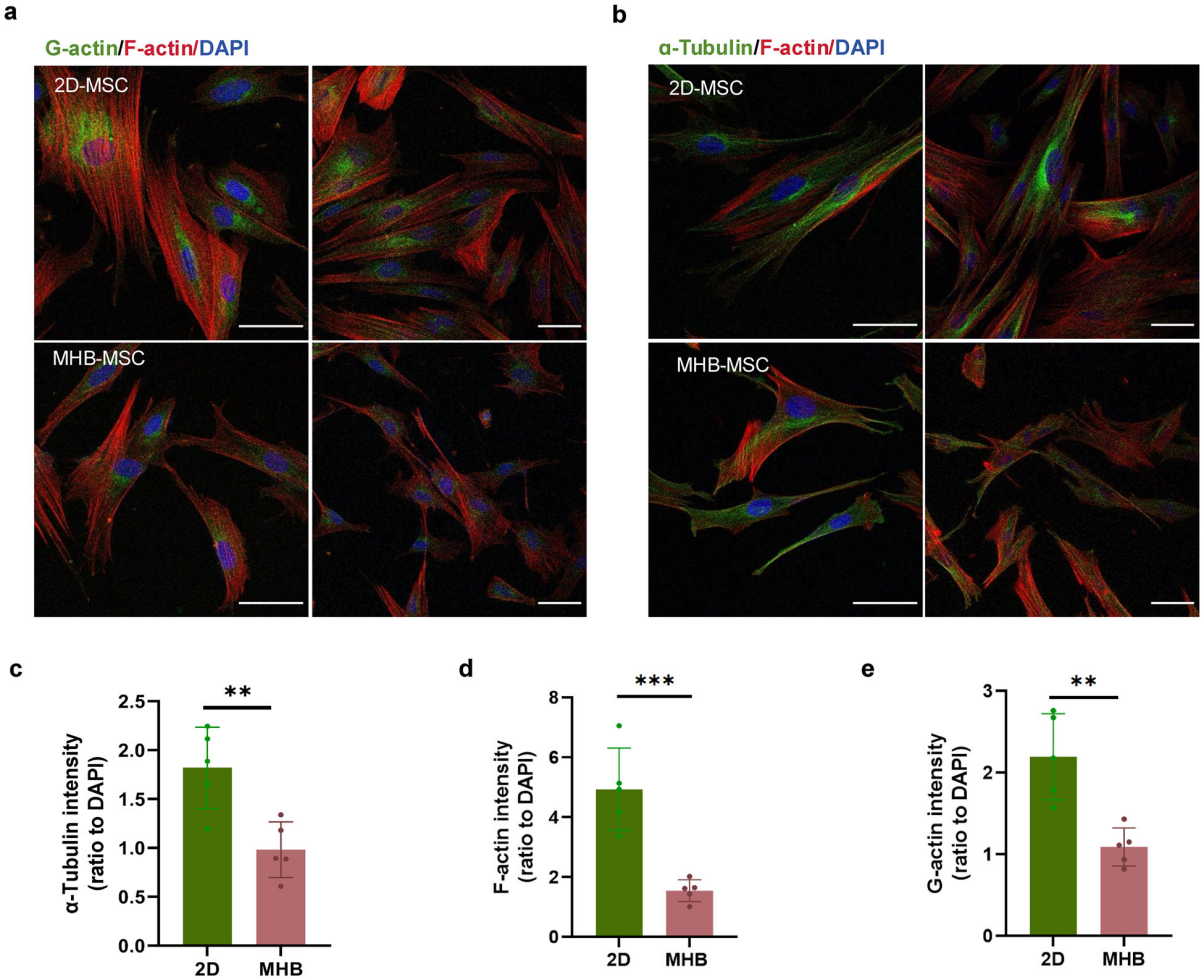
Cytoskeleton of hMSCs in 2D and MHB groups. (a and b) Immunostaining for G‐actin, F‐actin and ɑ‐tubulin of hMSC in 2D and MHB groups, Nuclei were labelled with DAPI. Higher magnification images were shown in the left panel. Scale bar, 50 µm. (c–e) Integrated intensity of ɑ‐tubulin (c), F‐actin (d) and G‐actin (e) was quantified and normalised to the intensity of DAPI. *n* = 5. *p < 0.05; **p < 0.01; ***p < 0.001; ****p < 0.0001.

It is known that 3D culture provides cells with a microenvironment with diverse physical features (Abdollahi 2021). The different mechanical properties affect the fate of hMSCs and are sensed and conducted by the actin cytoskeleton that underpins cell morphology (McKee and Chaudhry 2017). It was previously reported that 3D cultured hMSCs presented smaller cell size as a result of the relaxation of cytoskeleton tension (Zhou et al. 2017). And the remodelling of the cytoskeleton is closely related to the sorting of EVs (Liu et al. 2024). Thus, the investigation of how 3D microbead culture alters the cytoskeleton of hMSCs might provide significant clues on the augmented secretion of hMSC‐EVs. After being cultured in microbeads for 5 days, hMSCs were dissociated from beads and re‐adhered to culture plates. The organisation of F‐actin, G‐actin and α‐tubulin in hMSCs cultured in 2D condition and 3D microbead were first explored (Figure 3a,b). 2D cultured hMSCs exhibited more elongated F‐actin networks with branched and multidirectional F‐actin stress bundles at the cell edge as well as in the cell body, consistent with the flat morphology and larger size of hMSCs. However, microbead‐cultured hMSCs exhibited fewer F‐actin filament bundles and very thin filaments traversing the cell body, contributing to the smaller and uniformly spindle‐like morphology. Further, the integrated intensity of ɑ‐tubulin, F‐actin and G‐actin were calculated and normalised to the intensity of DAPI for comparison (Figure 3c–e). The fluorescent images show decreased levels of these markers in MHB groups compared with 2D group.

**FIGURE 4:**
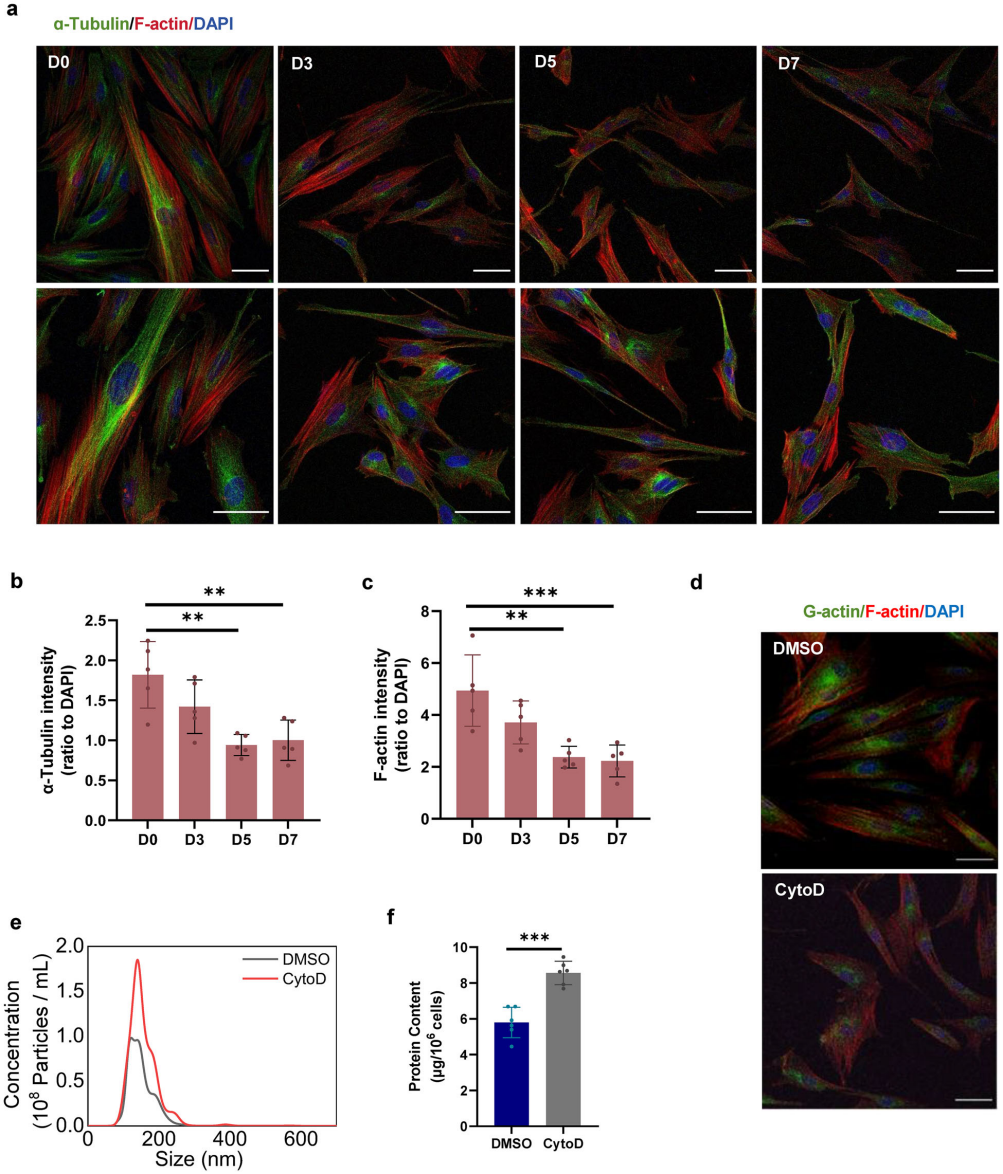
Decreasing actin polymerisation leads to the reduction of hMSC size and the increase of EV production. (a) Fluorescent images of F‐actin (red) and ɑ‐tubulin (green) in hMSCs retrieved from Matrigel microbeads at D0, D3, D5 and D7. Higher magnification images are shown in the lower panel. Scale bar, 50 µm. (b and c) Integrated intensity of ɑ‐tubulin (b) and F‐actin (c) was quantified and normalised to DAPI intensity at D0, D3, D5 and D7. *n* = 5 per group. (d) Immunofluorescence images of G‐actin and F‐actin in hMSCs treated with CytoD (0.8 µM) or vehicle control (0.1% DMSO) for 8 h. Scale bar, 50 µm. (e and f) NTA results of the concentration and size distribution (e) and protein content (f) of hMSC‐EVs. *p < 0.05; **p < 0.01; ***p < 0.001; ****p < 0.0001.

To monitor the long‐lasting effect of microbead culture on actin cytoskeleton alteration of hMSCs, hMSCs cultured in the MHB group were retrieved on Day 3, Day 5 and Day 7. hMSCs cultured under 2D conditions were considered the D0 group. According to the fluorescence images, hMSCs gradually presented smaller size and more spindle‐like morphology from Day 0 to Day 7, and these changes were most obvious before Day 5 (Figure 4a). Fluorescent staining images and quantification of fluorescent intensity exhibited that the level of both ɑ‐tubulin and F‐actin in MHB‐hMSCs gradually declined during the culture time, and the major reduction took place in the first 5 days (Figure 4b,c). This suggests that microbead culture releases actin cytoskeleton tension in hMSCs during the first 5 days.

Further, to investigate whether decreasing actin polymerisation causes the reduction of hMSC size and the increase of EV production, hMSCs were treated with Cytochalasin D (CytoD), a blocker of actin polymerisation and elongation, and adopted 0.1% DMSO as control. Results show that CytoD treatment dramatically reduced the level of F‐actin, G‐actin and cell size (Figure 4d). To determine whether increased EV secretion of hMSCs in microbead culture was caused by decreased cytoskeleton, EV adopted by CytoD was quantified by NTA and protein content comparison (Figure 4e,f). NTA results suggest that CytoD treatment markedly augmented EV secretion of hMSCs into the culture medium with all particle sizes ranging from 50 to 250 nm in diameter, compared with the control group. Consistently, our results suggest that actin depolymerisation in microbead culture might be an important mechanism for increasing vesicle secretion and the reduction of cell volume.

### 2.4 Angiogenic Potency of hMSC‐EVs Cultured in Microbeads and 2D Groups

**FIGURE 5:**
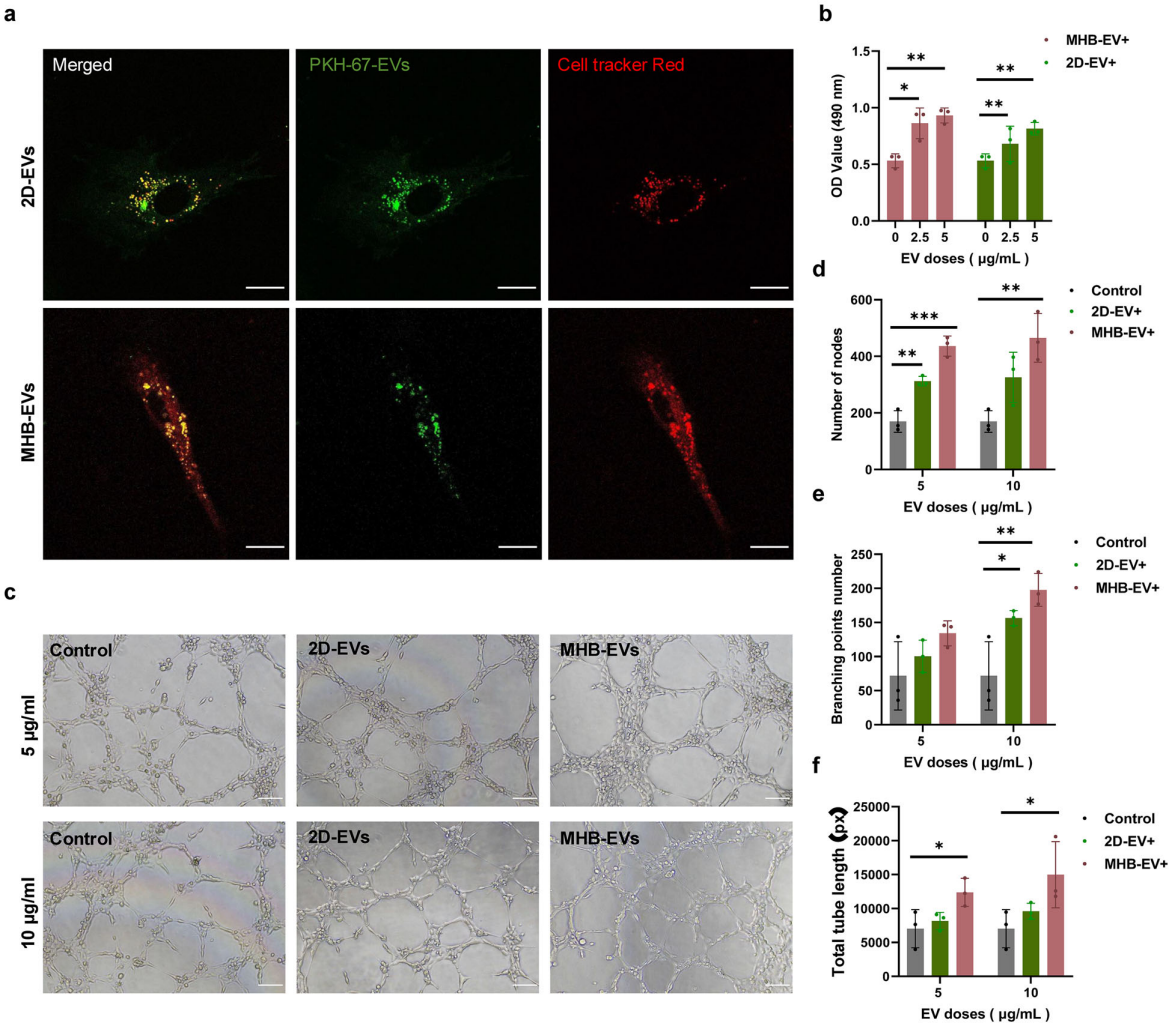
Angiogenic potency of hMSC‐EVs cultured in microbeads and 2D group. (a) Fluorescent images of cellular uptake of hMSCEVs (5 µg/mL) by HUVECs. Scale bar, 20 µm (b) OD values of MTT assays for cell proliferation of HUVEC with increased doses of 2D‐EVs and MHB‐EVs treatment for 48 h. *n* = 3. (c–f) The inducible capacity of hMSC‐EVs (5 and 10 µg/mL) for vascular tube formation. The control group represents untreated HUVECs. Scale bar, 200 µm. Number of nodes (d), branching points number (e) and total tube length (f) of the resulting tube formations were quantitatively compared. *n* =3.

To investigate the in vitro bioactivity of hMSC‐derived EVs, we used HUVEC to test the angiogenesis potential of hMSC‐EVs. The PKH67‐labelled EVs were added to the culture medium of Dil‐labelled HUVEC. After 48 h, EVs were observed in strong co‐localisation with HUVEC (Figure 5a), indicating that HUVEC had effectively internalised EVs derived from 2D and microbead groups. Moreover, the cell viability of treated HUVEC was promoted in an increasing EV dose‐dependent manner (0, 2.5 and 5 µg/mL) determined by MTT assay (Figure 5b).

To further evaluate the vascular tube formation effects of EV, 5 and 10 µg/mL of 2D‐EVs and MHB‐EVs were added to the culture medium of HUVECs seeded on Matrigel (Figure 5c). The tube formation was then quantified by measuring the number of nodes, branching points number and total tube length (Figure (5d–f). Compared to the control and 2D‐EVs groups, supplementation with MHB‐EVs enhanced tube formation in HUVECs.

**FIGURE 6:**
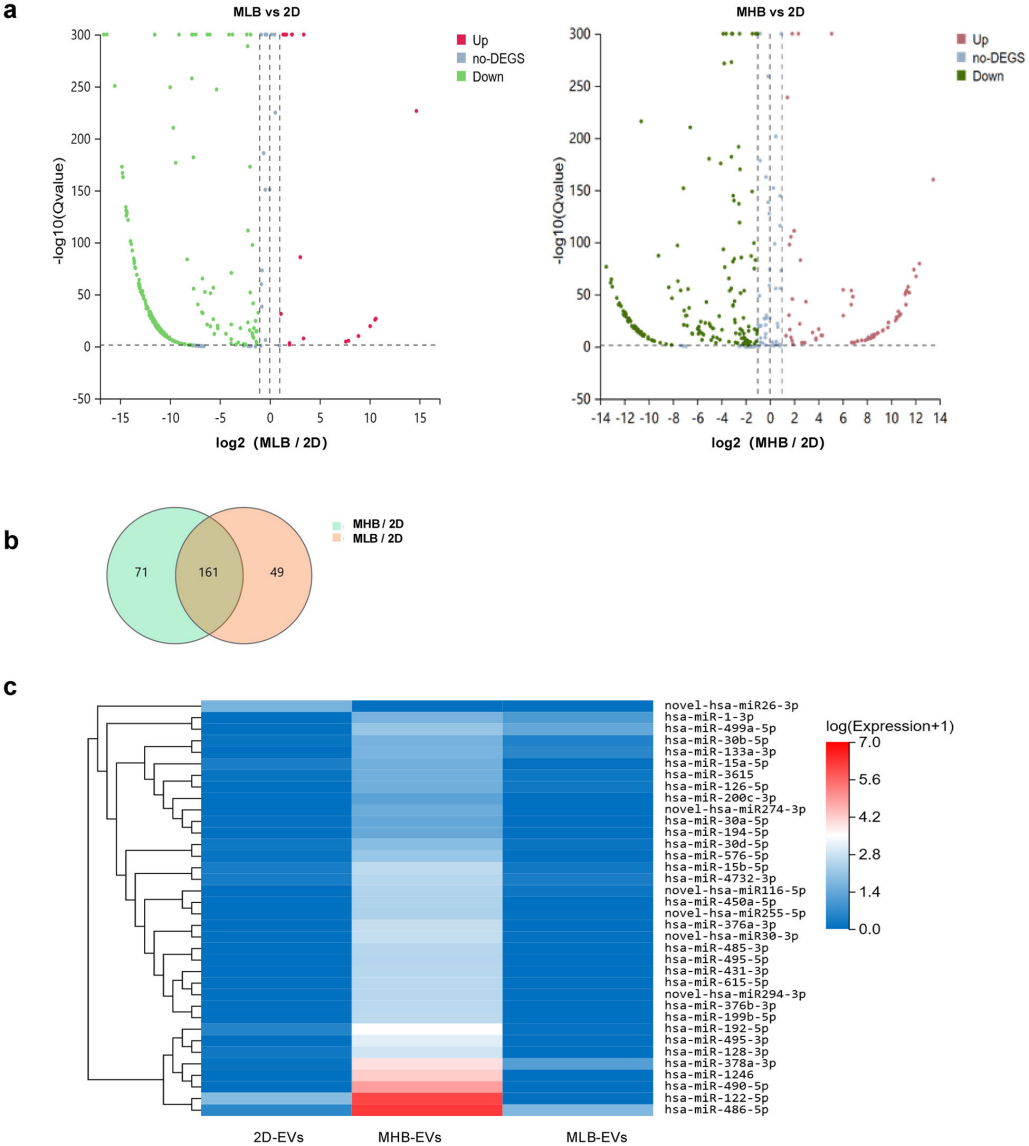
Comparison of miRNA profiles of EVs isolated from hMSC cultured in 2D, MHB and MLB conditions. (a) Volcano plot of differentially expressed miRNAs in MHB‐EVs and MLB‐EVs, both compared to 2D‐EVs. Upregulated miRNAs were selected when log2 (Fold Change) ≥1 and *Q*‐value ≤0.001; downregulated miRNAs were selected when log2 (Fold Change) ≤−1 and *Q*‐value ≤0.001. (b) Venn diagram showing the differentially expressed miRNAs from MHBEVs and MLB‐EVs, both compared to 2D‐EVs. (c) Heatmap of differentially expressed miRNAs related to angiogenesis in EVs derived from hMSCs cultured in 2D, MHB and MLB groups.

To further investigate the gene expression of EVs in the 2D and microbeads groups, miRNAs from both groups were sequenced. The volcano plots demonstrated that 30.71% of the differentially expressed miRNAs in MHB‐EVs and 10.00% in MLB‐EVs were upregulated compared to 2D‐EVs (Figure 6a). In addition, 71 and 49 differentially expressed miRNAs were found in MHB‐EVs and MLB‐EVs, respectively, compared to 2D‐EVs, and 161 miRNAs overlapped among the three groups (Figure 6b). To better exhibit the differences in miRNA expression, a classification heatmap of the miRNAs related to angiogenesis was performed (Figure 6c).

## 3 Discussion

hMSC‐EVs‐based therapies pose several challenges, including the large variation in therapeutic efficacies depending on source cell culture conditions and unscalable production, which hinders them from translating to clinical applications. This work proposes an experimental method for engineering hMSCs microbeads to build a more physiological‐related platform for highly efficient scaled‐up EV production, which can facilitate various applications of EV‐based therapies in clinical translation. Insights were also provided towards how microenvironmental cues improve and direct the hMSC secretory profile and how cellular spatial organisation and scaffolding materials affect the secretion, content and functions of hMSC‐EVs, which has important implications for designing targeted EVs for the treatment of specific diseases.

First, we used the bead‐jet printing platform based on our previous research design (Jiang et al. 2020; Xu et al. 2021; Cao et al. 2022), which can customise the printing of homogeneous cell microbeads, including precise control of the cell density in the microbeads, the size of the microbeads and the encapsulation material. By optimising the parameters of microfluidic printing, the system can effectively reduce mechanical damage and cell cost during preparation, laying the groundwork for exploring optimal EV production efficiency. The yield of hMSC‐EVs was increased 20‐fold in the MHB group compared to the 2D group. The stiffness of the extracellular matrix (ECM) plays a critical role in cell adhesion, proliferation, differentiation, migration and secretion (Raghav et al. 2022; Kim et al. 2022; Caldwell et al. 2020; Mao et al. 2016; Ylostalo 2020). Ultra‐soft scaffolds, such as hydrogels, are favoured for 3D cell culture due to their good ability to mimic the in vivo environment and promote cell growth and differentiation (Fan and Wang 2017; Lou and Mooney 2022; Price et al. 2017). Porous scaffolds have been reported to facilitate interactions of MSCs and promote their secretory properties (Caldwell et al. 2020). Biological material constitutes the cellular microenvironment, the interaction between the matrix and the cells affects the cellular behaviour and signalling pathways, which plays a pivotal role in regulating functions and secretion of MSC (Liu et al. 2024; Caldwell et al. 2020; Qazi et al. 2017). In this study, Matrigel was chosen as the scaffold material for cellular microbead encapsulation. Its rich collagen and fibronectin content is beneficial for interaction with cell surface integrins and facilitates the formation of ECM, thus enhancing cell‐cell signal communications (Wang et al. 2020). In addition to its composition, Matrigel provides suitable mechanical support for cells, and its ultra‐soft (<100 Pa) mechanical properties may also contribute to the secretion and function of hMSC‐EVs. In this study, ultrasoft Matrigel encapsulation has been observed to promote EV secretion, which is consistent with previous study (Qazi et al. 2017). Studies have shown that lower ECM stiffness would be more beneficial for prompting the yield and therapeutic efficacy of EVs (Liu et al. 2024; van de Wakker et al. 2024). For example, Rigid gel beads (e.g., gelatin methacrylate, alginate, etc., 1–10 kPa) limit cell growth and secretory behaviours, thus compromising their compatibility for EV production (Zhang et al. 2021). In addition, degradation of matrigel during the growth of hMSC microbeads may induce the structure of the microbeads to become loose, thus providing more space for cell growth and proliferation. In response to matrix degradation, cells may release specific signalling molecules via EV to influence the behaviour of surrounding cells, or increase EV secretion to regulate the extracellular environment.

Secondly, we investigated the effects of 3D microbead culture on the cytoskeleton organisation and EV secretion. We found that EVs from the MHB group had a smaller size compared to the 2D group, and the reduction in cytoskeleton tension was associated with increased EV secretion. Physical characteristics are important factors affecting the cytoskeleton. Actually, the fact that the size of an EV will be smaller in 3D environments than in 2D culture conditions was proved in a previous study. It has been found that 3D MSC is smaller than 2D MSC (Mo et al. 2018), and the 3D culture significantly enhanced the EV release by decreasing cytoskeletal tension, which reduced the size of MSCs and increased EV production (Cao et al. 2020). This difference in size may be due to the higher degree of cell‐cell interactions and lower ECM stiffness in the 3D environment, thus altering the cell morphology, cytoskeletal tension and EV secretion process. We, therefore, speculate that it is the 3D environment that affects the cellular skeleton and thus facilitates the process of EV efflux, whereby the massive secretion of EV leads to a smaller corresponding cell size in the MHB group. Then, we used CytoD to block the actin polymerisation and found that EV production would increase as cytoskeletal proteins decreased, thus further confirming the role of cytoskeletal tension in regulating EV secretion. This study emphasises the importance of cytoskeletal dynamics in regulating hMSC function and EV secretion in 3D culture.

Thirdly, we assessed the angiogenesis bioactivity of hMSC‐EVs. EVs from both 2D and microbead group were internalised by HUVECs and promoted the cell viability of HUVECs. MHB‐EVs showed enhanced angiogenesis compared to 2D‐EVs, indicated by increased tube formation. This suggests that EVs derived from 3D microbead cultures may have augmented potential for promoting angiogenesis. According to our previous study (Cao et al. 2022), microbead culture upregulated cell proliferation and cell migration via PI3K‐Akt signalling, and multiple secreted factors (HGF, VEGFA, FGF7, PDGFB) and receptors (EPOR, OSMR, TEK) in the signalling pathway were significantly enriched in hMSCs cultured in the MHB group. In this study, EVs extracted from the MHB group also exhibited up‐regulation of miRNAs associated with the PI3K‐Akt signalling pathway, for example, mir‐126‐5p, which was highly expressed in the MHB‐EV group has been shown to promote human umbilical vein endothelial cell (HUVEC) proliferation, migration and vasculature by targeting the PIK3R2‐mediated PI3K/Akt signalling pathway (Zhang et al. 2021). In addition to mir‐486‐5p, which was highly expressed in the MHB‐EV group, was also shown cardioprotective effects against ischemia‐reperfusion by inhibiting PDCD4 (Bei et al. 2022), suggesting that EVs derived from the MHB group have similar therapeutic functions in stimulating angiogenesis.

Apart from the aforementioned aspects, there are some directions worth further investigation. Firstly, our study focused on EV production per cell, concluding that microbead culture enhances hMSC‐derived functional EV efficiency. To balance culture time and the efficiency of EV production, a sustainable, repeatable EV collection system should be promoted. Our study has investigated the long‐term hMSC growth kinetics in microbead culture, and results showed that hMSC can still proliferate at Day 14. Thus, it is possible that EVs can be collected using the same batch of hMSCs continuously over a long period of time, adopting this strategy (Pasitka et al. 2024). In particular, previous studies using tangential flow filtration bioreactors validated that this continuous manufacturing can be used to scale up production at a reduced cost by more than 20 days of multiple harvests. In addition, the growth of adherent cells is limited by the surface area of the bioreactor, which is one of the challenges for the industrialisation of such bioreactors. Our system provides a microenvironment for MSCs in microbeads that may lay the foundation for their application. Secondly, in terms of EV characterisation and function, this study mainly used TEM and NTA to evaluate the morphology and yield of EV, in fact, in addition to these methods, flow cytometry, mass spectrometry and enzyme‐linked immunosorbent assay can be used to further characterise the marker and cargo of MSC‐EV (Kolenc and Maličev 2024; Simeone et al. 2020). Besides angiogenesis, EV is a major paracrine component of MSC, its other functions, such as immunosuppression, antifibrosis and promotion of regeneration in 3D culture environment, can be further explored. Thirdly, the RNA sequencing we performed identified differentially expressed genes of investigational significance between the 2D and 3D microbeads sets, but whether these genes play a role in regulating the yield or function of EVs remains an open question. Therefore, a focused investigation into the potential influence of these differential genes on EV production and function would be worthwhile.

## 4 Materials and Methods

### 4.1 Isolation and Characterisation of hMSCs

MSCs used in this study were isolated from human umbilical cord tissues. Human umbilical cord tissues were obtained from full‐term births after normal vaginal delivery at the delivery room of the Shenzhen Maternity and Child Healthcare Hospital. All the acquisitions of human samples were approved by the Ethic Committees of the Shenzhen Maternity and Child Healthcare Hospital, Project SFYLS [2020]061. Two donors (ref number 10011399 and 10016808) at Shenzhen Maternity and Child Healthcare Hospital volunteered to give the human umbilical cords for this study with informed consent. The tissues (length > 10 cm) were collected in a sterile jar containing PBS supplemented with 1% penicillin/streptomycin (P/S) (Gibco). The samples were transported on ice and processed within 12 h. Blood clots were removed by rinsing with PBS supplemented with 1% penicillin/streptomycin (P/S), the cords were cut into pieces (2–3 cm) and blood vessels were removed. The Wharton's jelly was chopped into fragments (1 mm^3^) by scissors and placed into cell culture flasks (25 cm^2^) for culture expansion at 37°C and 5% CO_2_. After the MSC crawled out of the tissue, cells were verified in vitro for the MSC markers CD90 and CD105, as well as the Osteogenic and as suggested by the International Society for Cell Therapies (ISCT). The data of MSC characterisation could be seen in our previous study (Cao et al. 2022).

### 4.2 Cell Culture

The MSCs were cultured in Dulbecco's modified Eagle's medium (DMEM)/F12 (Gibco, USA) containing 10% foetal bovine serum (FBS) (Gibco, USA) and 1% penicillin/streptomycin (P/S). In this study, MSCs between passages 3 and 10 (P3–P10) were used. Umbilical Vein Endothelial Cells (HUVEC) were cultured in endothelial growth medium (EGM) from P1 to P8. Cells were both maintained in a humidified CO_2_ incubator at 37°C, and cell culture media was changed and refreshed every 2 or 3 days.

### 4.3 Formulation of hMSC‐Microbeads

A customised bead‐jet printing platform has been designed based on our previous work (Jiang et al. 2020; Xu et al. 2021; Cao et al. 2022). The system consisted of two modules operating in synchronisation, a customised droplet microfluidics module (M1) for microbead printing and formulation and a bead collection module (M2) (Figure 1a). M1 module consists of two fluid injection pumps (LEAD FLUID) and two pipelines, the oil pipeline and the cell suspension pipeline. Before printing, monodisperse cell‐laden droplets can be encapsulated by different biomaterials. In this study, Matrigel (Corning, USA) was used to mix and encapsulate hMSC in advance. To fabricate Matrigel‐hMSC‐beads, the Matrigel‐hMSC‐liquid phase at 4°C was loaded into a 1 mL syringe (Yuekang) and then connected with the pump and cell suspension pipeline (the flow rate was set at 20 µL/min). The HFE7000 oil was loaded in a 10 mL syringe (Yuekang) connected to another fluid injection pump and oil pipeline (the flow rate was set at 300 µL/min). Through a 3‐way PDMS cube, the Matrigel‐hMSC‐liquid phase will be sheared into monodisperse Matrigel‐hMSC‐beads by a constant stream of HFE7000 oil. The tube outlet of M1 is connected to a 3‐port connector (PDMS shaped) with a side channel (width = 1 mm), which is connected with a long collection tube (Woer, inner diameter (ID) = 0.56 mm). When the suspension of Matrigel‐hMSCs passes through the 3‐way PDMS cube, it will be sheared by the oil in the tubing into homogeneous cellular microbeads, which will be collected in a long connecting tube and then gelled in an M2 module at 37°C for 30 min. At last, the microbeads will be injected through a vertical channel onto the culture plate by the compressed air flow at the intersection point of the nozzle. This method was highly productive for the large‐scale formation of size‐controlled cell microbeads, and it only took less than 10 min to fabricate over 1000 microbeads. By varying the number of cells in a fixed volume of Matrigel, hMSC‐microbeads with different cell densities can be produced consistently. The cell numbers in the MHB and MLB groups were 1500 and 300 cells/bead, respectively. The size of the microbeads depends on the inner diameter of the tube (500 µm/beads in this study).

### 4.4 Collection and Isolation of hMSC‐EVs

In the microbead group, the printed hMSCs microbeads were cultured for 5 days in 12‐well plates with 1 mL culture medium and 50 microbeads/well. In 2D groups, hMSCs were seeded on Day 2 and cultured until Day 5. Next, the complete condition medium in all groups was replaced with EV‐depleted condition medium (only FBS were replaced with EV‐depleted FBS [C38010100, Vivacell]) and culture for another 2 days. Then EVs were collected from the medium of all groups by ultracentrifugation for EV isolation. The collected medium was centrifuged at 2000 × *g* for 30 min, then at 10000 × *g* for 30 min and the supernatant was collected and subjected to a 0.22‐µm filter. Afterwards, the supernatant was centrifuged at 120,000 × *g* for 70 min at 4°C and pellets were collected and subjected to another centrifugation at 120,000 × *g* for 70 min at 4°C. The final EV pellet was resuspended in PBS and stored at −80°C.

### 4.5 Characterisation of EV (Production and Morphology)

NTA was adopted to assess the EV concentration and size distribution by NanoSight NS300 instrument (Malvern, Worcestershire, UK) with a scientific CMOS sensor. Samples were diluted in PBS buffer and three 40 s videos were recorded at the detection threshold 7. Data were displayed on NTA software 3.0, and original data was analysed using Image J (version 1.52). NTA results of particle concentration in EV samples derived from identical initial cell numbers were first compared, and the total number of EVs isolated in each group was normalised to the total number of cells producing these EVs (cells at Day 7) (Figure 2f). The BCA Protein Assay Kit (23227, Thermo Fisher Scientific) was used for the determination of the protein content of EVs. RNA of EVs were isolated using a DNA/RNA Isolation kit (TIANGEN, China) and the concentration was determined using the Nanodrop 2000 (Thermo, USA).

TEM was adopted to examine the morphology of EVs. Purified EVs were first fixed with 2% Paraformaldehyde (PFA) for 5 min, and 10 µL of EV sample was deposited on the formvar‐carbon‐coated grids for 2–3 min. Then, the grid was stained with 3% uranyl acetate for 1 min at room temperature and then rinsed in water to remove the excess staining solution. A filter paper was used to gently remove the extra solution on the grid edge. Then, Tecnai G2 Spirit TEM was used to examine the morphology of EVs at 80 kV.

### 4.6 Live and Dead Cells Staining and Counting

LIVE/DEAD staining kit (Yeasen, China) was adopted to assess the viability of cells in microbeads. After washing hMSC‐laden microbeads three times with 1×PBS buffer, live cells were stained by 2.0×10‐6 M Calcein AM and dead cells were stained by 4.5×10‐6 M PI for 30 min. Then, cells were gently washed by 1×PBS buffer. The fluorescent microscope (Nikon Eclipse Ts2r) was used for capturing all microscopic images, and the number of live (green) and dead (red) cells in the images was calculated to quantify the viability ratio.

### 4.7 Immunofluorescence Staining of hMSCs

hMSCs were extracted from the MHB group on Day 3, Day 5 and Day 7, respectively. hMSCs cultured under 2D conditions were considered the D0 group. Those retrieved hMSCs were all dissociated from microbeads or 2D plate culture and then were cultured for 48 h in 12‐well glass bottom plates and then fixed using 4.0% PFA for 1 h at room temperature. After washing hMSCs three times with PBS buffer, cells were permeabilised with 0.5% Triton X‐100 for 30 min and then incubated with 5% BSA for 1 h at room temperature. Then, hMSCs were stained by Alexa Flura 594‐ phalloidin (ThermoFisher, A12381) for visualisation of F‐actin, Alexa Fluor 488 DNase I (ThermoFisher, D12371) for visualisation of G‐actin, Tubulin‐Tracker Green (Beyotime, C1051S) for visualisation of α‐tubulin. DAPI was used to stain cell nuclei. Fluorescent images were captured by confocal microscope (Nikon Eclipse Ts2r) and analysed using ImageJ (version 1.52).

### 4.8 Tracking Cellular Uptake of EVs

To visualise the cellular uptake of EVs, we first labelled 2D‐EVs and MHB‐EVs using PKH67 dye following the manufacturer's protocol. First, EVs were diluted using Diluent C, and then incubated with PKH67 dye for 5 min at room temperature. EV‐depleted FBS was used to stop the staining reaction, and ultracentrifugation was adopted to remove the superfluous dyes with PKH67‐labelled EVs that remained as pellets. Then, EV pellets were resuspended in 1× PBS by gentle pipetting. Dil cell membrane Tracker Red (MB4240‐1, Meilunbio) was used to label HUVEC. 10 µg/mL PKH67‐ labelled MHG‐EVs, MLB‐EVs and MHB‐EVs were supplemented in the culture media (containing EV‐depleted FBS) of HUVEC for 2 days. The visualisation of the colocalisation of EVs and cells was obtained by a confocal microscope (Nikon A1) at 100× magnification.

### 4.9 Western Blot

RIPA buffer (MCE) containing protease and phosphatase inhibitors (MCE) was used to lyse EVs derived from different groups. The BCA Protein Assay Kit (23227, Thermo Fisher Scientific) was adopted for determining protein concentrations, and equal amounts of total proteins in each group were loaded for gel electrophoresis. Briefly, 10% SDS‐polyacrylamide gels were used to separate proteins, and separated proteins were electrophoretically transferred to a PVDF membrane (Millipore). The membrane was incubated overnight at 4°C with primary antibodies after blocking. Primary antibodies include, antiHsp70 (1:1000, Abcam, ab181606), anti‐TSG101 (1:1000, Abcam, ab125011) and antiCD63 (1:1000, Abcam, ab134045). Then, the membranes were incubated with the secondary antibody: anti‐mouse IgG H&L (HRP) (1:5000; Abcam, ab6789) at room temperature. Band visualisation was performed using the Chemiluminescent Western Blot detection kit (4AW012‐1000, 4A Biotech).

### 4.10 Assessment of Cell Viability and Proliferation by MTT

HUVEC cells were inoculated into 96‐well plate at 2500 cells/well in 100 µL volume and cultured overnight at 37°C with 5% CO2. 2.5 and 5 µg/mL of 2D‐EVs and MHB‐EVs were supplemented in the culture media of HUVEC for 48 h. An identical volume of PBS buffer was supplemented as the control group. After 48 h, MTT assay (Sigma‐Aldrich) was used to assess cell growth. MTT reagent (5 mg/mL) was added to each well in the cell culture media, and cells were further cultured at 37°C in the dark for 4 h. Then, Dimethyl Sulfoxide (DMSO) was supplemented for dissolving formazan crystals and 50 µL of the solution from each well was transferred to a new 96 well plates. Then, the optical density (OD) value at 490 nm was measured by Synergy H1 microplate reader (Bio‐Teck, Winooski, VT, USA).

### 4.11 Tube Formation Assay

Passage 3–5 HUVECs were seeded on a Matrigel‐coated plate in EGM. 5 and 10 µg/mL of 2D‐EVs, MHB‐EVs were added to the HUVECs. The control group of untreated HUVECs was also included. The resulting tube formation was observed by microscope. Tube formation of the HUVECs was observed 6 h after treatment. The tube length, branching points number and tube numbers of the resulting tube formations were quantitatively compared using WIMASIS Software.

### 4.12 RNA‐Seq Analysis

The small RNA sequencing of EVs was performed by BGI‐Shenzhen, China. Total RNA was extracted from EVs derived from different groups using miRNeasy Micro Kit (Qiagen, Germantown, MD, USA), according to the manufacturer's manual. Total RNA was qualified and quantified using a NanoDrop and Agilent 2100 bioanalyzer (Thermo Fisher Scientific, MA, USA). The miRNA library construction and sequencing process were carried out according to the company's protocol on DNBSEQ platform. DESeq2 package was used to identify differential expression genes. The value of gene expression was calculated and normalised as Fragments Per Kilobase of transcript per Million mapped reads (FPKM). Generally, the function of *Q* value ≤ 0.05 is regarded as a significant enrichment. The small RNA‐seq data described in this thesis are available at Gene Expression Omnibus (GEO) under accession GSE215294.

### 4.13 Statistical Analysis

All the statistical analysis was performed by Origin 2021b (OriginLab Co, Northampton, MA, USA) and Prism 6 (GraphPad, Inc.) software. Significant differences between parallel groups were calculated using unpaired two‐tailed Student's *t*‐test (comparing two experimental groups) or one‐way ANOVA (more than two groups involved) following Tukey's multiple comparisons tests. Unless indicated otherwise, the data are represented as mean ± SD and a significant difference was considered as *p* < 0.04. All representative fluorescence images, TEM images and bright field images shown in the main text were repeated at least three times independently with similar results.

## Author Contributions

**Jiayi Tan**: Conceptualization, investigation, data interpretation and manuscript writing. **Yunxia Hu**: conceptualisation, investigation, formal analysis. **Lijuan Zheng**: data curation and writing–original draft. **Zheng Zheng, Mali Fu and Haiying Peng**: resources and supervision. **Shaohua Ma**: conceptualisation, funding acquisition and supervision. All authors have read, revised and approved the content of the manuscript.

## Conflicts of Interest

The authors declare no conflicts of interest.

## Data Availability

The original data relevant to the study are included in the article or uploaded as online supplemental information.
