# Correction to Microbead Encapsulation Strategy for Efficient Production of Extracellular Vesicles Derived From Human Mesenchymal Stem Cells

**DOI:** 10.1002/jev2.70217

**Published:** 2025-12-19

Tan, J., Y. Hu, L. Zheng, et al. 2025. “Microbead Encapsulation Strategy for Efficient Production of Extracellular Vesicles Derived From Human Mesenchymal Stem Cells.” *Journal of Extracellular Vesicles* 14, no. 4: e70053. https://doi.org/10.1002/jev2.70053.

The footnote of this article has been updated to read Jiayi Tan, Yunxia Hu, and Lijuan Zheng contributed equally to this work. This was left out of an earlier corrected version of this article. The online version of this article has been corrected.

We apologize for this error.

